# Supplementary material for: Prediction of life stress on athletes’ burnout: the dual role of perceived stress
Source: PeerJ. 2018 Jan 15;6:e4213. doi: 10.7717/peerj.4213 (PMC5772382; doi:10.7717/peerj.4213)
Supplement: Supplemental Information 1 [file peerj-06-4213-s001.docx]

| 各位同學您好：  　　本人正在進行運動員壓力。請你協助填寫問卷，並就每一問題題目，圈選出你的個人最獨到經驗或看法。問卷沒有對或錯，保密且不記名，所以不會影響你或任何人的權益。請放心作答。請圈選出代表您最真實感受的答案。  　　　　　　　　　　　　　　　　　　 　　　　　　　　　　　　　　　　　　　　　中國文化大學 　盧俊宏 敬上 |
| --- |

**【基本資料】**

一、 性別 € 男 € 女

二、 年齡 _______ 歲

三、 身高 _______ 公分 ; 體重 _______ 公斤

四、運動專長項目是 ；從事這專項運動訓練與比賽共約 年

五、每天訓練 共 小時；每週訓練 ______ 天

六、近一年來參加比賽最高等級，請打勾： （國際賽） ； （全國賽）

（大專比賽）； （縣市比賽） ； （校際比賽）

七、就讀學校：_________；年級 _________

**CSE量表**

| 身為運動員，當事情不太順利，或者您有麻煩時， **您**有**多大把握**使用下列26方法來克服困難 …………  (圈選出代表您個人最真實感受的答案) | **完全沒把握** |  |  |  |  | **普通把握** |  |  |  |  | **絕對有把握** |
| --- | --- | --- | --- | --- | --- | --- | --- | --- | --- | --- | --- |
| **1.** 把煩人的問題切割成很小的部份來解決 | 0 | 1 | 2 | 3 | 4 | 5 | 6 | 7 | 8 | 9 | 10 |
| **2.** 想看看什麼可以改變，什麼不可以改變 | 0 | 1 | 2 | 3 | 4 | 5 | 6 | 7 | 8 | 9 | 10 |
| **3.** 讓不愉快的想法遠離我 | 0 | 1 | 2 | 3 | 4 | 5 | 6 | 7 | 8 | 9 | 10 |
| **4.** 讓自己心裡不再想起這些不愉快念頭 | 0 | 1 | 2 | 3 | 4 | 5 | 6 | 7 | 8 | 9 | 10 |
| **5.** 找朋友幫助您解決問題 | 0 | 1 | 2 | 3 | 4 | 5 | 6 | 7 | 8 | 9 | 10 |
| **6.** 找朋友或家人尋求安慰 | 0 | 1 | 2 | 3 | 4 | 5 | 6 | 7 | 8 | 9 | 10 |
| **7.** 在腦中構思一個計畫，然後依計畫來應付 | 0 | 1 | 2 | 3 | 4 | 5 | 6 | 7 | 8 | 9 | 10 |
| **8.** 當事情變得大條時，找各種可能的解決方法 | 0 | 1 | 2 | 3 | 4 | 5 | 6 | 7 | 8 | 9 | 10 |
| **9.** 盡量讓自己不要被不愉快想法激怒 | 0 | 1 | 2 | 3 | 4 | 5 | 6 | 7 | 8 | 9 | 10 |
| **10.** 結交新朋友 | 0 | 1 | 2 | 3 | 4 | 5 | 6 | 7 | 8 | 9 | 10 |
| **11.** 每一次考慮到問題的一部份，一步一步來 | 0 | 1 | 2 | 3 | 4 | 5 | 6 | 7 | 8 | 9 | 10 |
| **12.** 找出最困難部份的解決方案 | 0 | 1 | 2 | 3 | 4 | 5 | 6 | 7 | 8 | 9 | 10 |
| **13.** 想辦法遠離悲傷情緒 | 0 | 1 | 2 | 3 | 4 | 5 | 6 | 7 | 8 | 9 | 10 |
| **14.** 避免在困難時，更陷入低潮 | 0 | 1 | 2 | 3 | 4 | 5 | 6 | 7 | 8 | 9 | 10 |
| **15.** 當您失意時，做一些對你有正面的事情 | 0 | 1 | 2 | 3 | 4 | 5 | 6 | 7 | 8 | 9 | 10 |
| **16.** 尋找社區機構或資源支持 | 0 | 1 | 2 | 3 | 4 | 5 | 6 | 7 | 8 | 9 | 10 |
| **17.** 在壓力下避免衝動地解決問題 | 0 | 1 | 2 | 3 | 4 | 5 | 6 | 7 | 8 | 9 | 10 |
| **18.** 如果第一個方案不佳，再找其他解決方法 | 0 | 1 | 2 | 3 | 4 | 5 | 6 | 7 | 8 | 9 | 10 |
| **19.** 對自己正面的談話 | 0 | 1 | 2 | 3 | 4 | 5 | 6 | 7 | 8 | 9 | 10 |
| **20.** 在負面情境時，尋找一些正面的事物 | 0 | 1 | 2 | 3 | 4 | 5 | 6 | 7 | 8 | 9 | 10 |
| **21.** 不讓自己得情緒孤單 | 0 | 1 | 2 | 3 | 4 | 5 | 6 | 7 | 8 | 9 | 10 |
| **22.** 堅持原則對抗您想要的 | 0 | 1 | 2 | 3 | 4 | 5 | 6 | 7 | 8 | 9 | 10 |
| **23.** 在激烈爭辯中，站在對方立場著想 | 0 | 1 | 2 | 3 | 4 | 5 | 6 | 7 | 8 | 9 | 10 |
| **24.** 尋找新的嗜好或休閒活動 | 0 | 1 | 2 | 3 | 4 | 5 | 6 | 7 | 8 | 9 | 10 |
| **25.** 想像一個愉快的活動或地點 | 0 | 1 | 2 | 3 | 4 | 5 | 6 | 7 | 8 | 9 | 10 |
| **26.** 祈禱或冥想靜坐 | 0 | 1 | 2 | 3 | 4 | 5 | 6 | 7 | 8 | 9 | 10 |

**運動組織壓力指標 (OSI-SP)**

| 在過去的一個月裡，在競賽情境，我體驗到下列組織壓力，並且造成影響… (圈選出代表您個人最真實感受的答案) | | **完全沒有** | **大部分沒有** | **很少有** | **有時如此** | **大部分如此** | **總是如此** |
| --- | --- | --- | --- | --- | --- | --- | --- |
|  | …我在隊上的責任 | 1 | 2 | 3 | 4 | 5 | 6 |
|  | …我和教練的關係 | 1 | 2 | 3 | 4 | 5 | 6 |
|  | …我從事運動項目的規則/規定 | 1 | 2 | 3 | 4 | 5 | 6 |
|  | …我教練的個性 | 1 | 2 | 3 | 4 | 5 | 6 |
|  | …訓練/比賽的住宿環境 | 1 | 2 | 3 | 4 | 5 | 6 |
|  | …訓練/比賽的場地 | 1 | 2 | 3 | 4 | 5 | 6 |
|  | …管理和控制這項運動的組織 | 1 | 2 | 3 | 4 | 5 | 6 |
|  | …我所屬團隊的整體氣氛 | 1 | 2 | 3 | 4 | 5 | 6 |
|  | …我們隊上的選拔過程 | 1 | 2 | 3 | 4 | 5 | 6 |
|  | …我隊友的態度 | 1 | 2 | 3 | 4 | 5 | 6 |
|  | …看我表現的觀衆 | 1 | 2 | 3 | 4 | 5 | 6 |
|  | …我吃的東西 | 1 | 2 | 3 | 4 | 5 | 6 |
|  | …隊上共同的想法 | 1 | 2 | 3 | 4 | 5 | 6 |
|  | …媒體對我的報導 | 1 | 2 | 3 | 4 | 5 | 6 |
|  | …取得代表參賽的選拔制度 | 1 | 2 | 3 | 4 | 5 | 6 |
|  | …我的訓練課表 | 1 | 2 | 3 | 4 | 5 | 6 |
|  | …我所參與比賽的組織 | 1 | 2 | 3 | 4 | 5 | 6 |
|  | …受傷 | 1 | 2 | 3 | 4 | 5 | 6 |
|  | …這項運動的補助經費 | 1 | 2 | 3 | 4 | 5 | 6 |
|  | …我的運動生涯發展 | 1 | 2 | 3 | 4 | 5 | 6 |
|  | …我的運動中所使用的科技器材 | 1 | 2 | 3 | 4 | 5 | 6 |
|  | …訓練或比賽的旅程 | 1 | 2 | 3 | 4 | 5 | 6 |
|  | ……我的（體能/技術/比賽）目標 | 1 | 2 | 3 | 4 | 5 | 6 |

**CSALSS 量表**

| 在過去的一個月裡，我體驗到下列生活壓力， 並且造成影響… | | **完全沒有** | **大部分沒有** | **很少有** | **有時如此** | **大部分如此** | **總是如此** |
| --- | --- | --- | --- | --- | --- | --- | --- |
| **1.** | 運動傷害無法痊癒 | 1 | 2 | 3 | 4 | 5 | 6 |
| **2.** | 競賽表現不穩定 | 1 | 2 | 3 | 4 | 5 | 6 |
| **3.** | 與教練關係不佳 | 1 | 2 | 3 | 4 | 5 | 6 |
| **4.** | 不適應現在的訓練計畫 | 1 | 2 | 3 | 4 | 5 | 6 |
| **5.** | 不會處理人際關係 | 1 | 2 | 3 | 4 | 5 | 6 |
| **6.** | 沒有機會認識戀愛對象 | 1 | 2 | 3 | 4 | 5 | 6 |
| **7.** | 家人對我的期望太高 | 1 | 2 | 3 | 4 | 5 | 6 |
| **8.** | 沒有動機念書 | 1 | 2 | 3 | 4 | 5 | 6 |
| **9.** | 經常運動傷害 | 1 | 2 | 3 | 4 | 5 | 6 |
| **10.** | 表現不佳影響團隊成績 | 1 | 2 | 3 | 4 | 5 | 6 |
| **11.** | 教練偏袒隊上某些人 | 1 | 2 | 3 | 4 | 5 | 6 |
| **12.** | 訓練方式對我沒有幫助 | 1 | 2 | 3 | 4 | 5 | 6 |
| **13.** | 沒有朋友 | 1 | 2 | 3 | 4 | 5 | 6 |
| **14.** | 遇見喜歡的對象不敢表示 | 1 | 2 | 3 | 4 | 5 | 6 |
| **15.** | 家中遭遇困境 | 1 | 2 | 3 | 4 | 5 | 6 |
| **16.** | 不知道如何準備考試 | 1 | 2 | 3 | 4 | 5 | 6 |
| **17.** | 運動傷害復原速度慢 | 1 | 2 | 3 | 4 | 5 | 6 |
| **18.** | 運動表現不佳遭到淘汰 | 1 | 2 | 3 | 4 | 5 | 6 |
| **19.** | 教練對我有偏見 | 1 | 2 | 3 | 4 | 5 | 6 |
| **20.** | 訓練負荷太重 | 1 | 2 | 3 | 4 | 5 | 6 |
| **21.** | 別人不喜歡與我交朋友 | 1 | 2 | 3 | 4 | 5 | 6 |
| **22.** | 不知道該如何與伴侶相處 | 1 | 2 | 3 | 4 | 5 | 6 |
| **23.** | 不知道如何與家人溝通 | 1 | 2 | 3 | 4 | 5 | 6 |
| **24.** | 不知道怎麼有效率的學習課業 | 1 | 2 | 3 | 4 | 5 | 6 |

**PSS 量表**

| 在過去的一個月裡，你是否體驗到下列事件， 並且感到心煩… | | **從來沒有** | **大部分沒有** | **有時如此** | **大部分如此** | **總是如此** |
| --- | --- | --- | --- | --- | --- | --- |
|  | 因為某事不如預期而感到沮喪？ | 0 | 1 | 2 | 3 | 4 |
|  | 覺得無法控制人生中某些重要的事？ | 0 | 1 | 2 | 3 | 4 |
|  | 覺得緊張/有壓力？ | 0 | 1 | 2 | 3 | 4 |
|  | 能夠有效解決煩人的生活問題？ | 0 | 1 | 2 | 3 | 4 |
|  | 覺得可以有效處理生活中重要的問題？ | 0 | 1 | 2 | 3 | 4 |
|  | 對於自己處理個人問題感到有信心？ | 0 | 1 | 2 | 3 | 4 |
|  | 覺得事情會照著你想要的方式走？ | 0 | 1 | 2 | 3 | 4 |
|  | 發現沒有辦法處理你必須要做的事？ | 0 | 1 | 2 | 3 | 4 |
|  | 覺得自己有能力控制生活中困擾的事情？ | 0 | 1 | 2 | 3 | 4 |
|  | 覺得自己是最優秀的？ | 0 | 1 | 2 | 3 | 4 |
|  | 因為某事不受你控制而感到生氣？ | 0 | 1 | 2 | 3 | 4 |
|  | 常思考某些事情是你必需要完成的？ | 0 | 1 | 2 | 3 | 4 |
|  | 覺得自己有能力控制自己的時間？ | 0 | 1 | 2 | 3 | 4 |
|  | 覺得有許多困難無法去克服？ | 0 | 1 | 2 | 3 | 4 |

**ABQ量表**

| 對於**您**現在從事的這項運動， 您是否覺得…… | | **從來沒有** | **大部分沒有** | **很少有** | **有時如此** | **大部分如此** | **總是如此** |
| --- | --- | --- | --- | --- | --- | --- | --- |
| **1.** | 這項運動中，我完成了許多有價值的事 | 1 | 2 | 3 | 4 | 5 | 6 |
| **2.** | 由於訓練之故，我覺得很累，以致我沒有多餘精力從事  其他事物 | 1 | 2 | 3 | 4 | 5 | 6 |
| **3.** | 我耗費在這項運動上的努力，不如把它用在其它事物上 | 1 | 2 | 3 | 4 | 5 | 6 |
| **4.** | 因為從事這項運動之故，我覺得非常累 | 1 | 2 | 3 | 4 | 5 | 6 |
| **5.** | 我覺得在這項運動中並沒有多大成就 | 1 | 2 | 3 | 4 | 5 | 6 |
| **6.** | 我覺得我不像過去那麼在乎這項運動的成績表現 | 1 | 2 | 3 | 4 | 5 | 6 |
| **7.** | 在這項運動中，我覺得我並沒有把我的潛能完全發揮  出來 | 1 | 2 | 3 | 4 | 5 | 6 |
| **8.** | 我覺得我被這項運動榨乾了 | 1 | 2 | 3 | 4 | 5 | 6 |
| **9.** | 我覺得不像過去那麼喜歡這項運動 | 1 | 2 | 3 | 4 | 5 | 6 |
| **10.** | 由於這項運動之故，我覺得身體很疲倦 | 1 | 2 | 3 | 4 | 5 | 6 |
| **11.** | 我覺得我不像過去那般在乎在這項運動上的成功 | 1 | 2 | 3 | 4 | 5 | 6 |
| **12.** | 由於這項運動精神和身體上的要求，我覺得我已經耗  盡了 | 1 | 2 | 3 | 4 | 5 | 6 |
| **13.** | 似乎不管我如何做，我覺得我無法到達我應有的表現 | 1 | 2 | 3 | 4 | 5 | 6 |
| **14.** | 在這項運動中，我覺得我相當成功 | 1 | 2 | 3 | 4 | 5 | 6 |
| **15.** | 我覺得這項運動沒有什麼了不起 | 1 | 2 | 3 | 4 | 5 | 6 |
