# Supplementary material for: Prediction of life stress on athletes’ burnout: the dual role of perceived stress
Source: PeerJ. 2018 Jan 15;6:e4213. doi: 10.7717/peerj.4213 (PMC5772382; doi:10.7717/peerj.4213)
Supplement: Supplemental Information 3 [file peerj-06-4213-s003.docx]

**College Student-Athletes’ Life Stress Scale (CSALSS)**

Directions: Below are 24 statements that describe something that annoys/bothers you or makes you uncomfortable in your daily life as a college student-athlete. Please read each one carefully and circle the number that indicates how often you experience it. Your answers are absolutely confidential.

1. **Never; 2. Rarely; 3.Sometimes; 4.Quite often; 5. Very often; 6. Always**

| I l am annoyed by my injury because it has still not yet fully recovered. | 1 | 2 | 3 | 4 | 5 |  | 6 |
| --- | --- | --- | --- | --- | --- | --- | --- |
| 2 I worry about my unstable competitive performance. | 1 | 2 | 3 | 4 | 5 |  | 6 |
| 3 I am annoyed by my disappointing relationship with my coach. |  | 2 | 3 | 4 | 5 |  | 6 |
| 4 I am annoyed with the training program now. | 1 | 2 | 3 | 4 | 5 |  | 6 |
| 5 I am bothered by poor social skills in handling interpersonal relationships. | 1 | 2 | 3 | 4 | 5 |  | 6 |
| 6 I am annoyed with not finding time to encounter romantic partners. | 1 | 2 | 3 | 4 | 5 |  | 6 |
| 7 I am annoyed by my parents' high expectations. | 1 | 2 | 3 | 4 | 5 |  | 6 |
| 8 I am bothered by a lack of motivation for academic learning. | 1 | 2 | 3 | 4 | 5 |  | 6 |
| 9 I worry about being frequently injured. | 1 | 2 | 3 | 4 | 5 |  | 6 |
| 10 I worry about dragging my team down. | 1 | 2 | 3 | 4 | 5 |  | 6 |
| 11 I am annoyed by my coach's preference for some teammates. | 1 | 2 | 3 | 4 | 5 |  | 6 |
| 12 I worry that my training is not beneficial to my performance. | 1 | 2 | 3 | 4 | 5 |  | 6 |
| 13 I am annoyed with being friendless. | 1 | 2 | 3 | 4 | 5 |  | 6 |
| 14 I am annoyed with being too shy to express myself when I encounter someone I love. |  | 2 | 3 | 4 | 5 |  | 6 |
| 15 l am bothered by difficult situations in my family. | 1 | 2 | 3 | 4 | 5 |  | 6 |
| 16 I am annoyed when preparing for exams. | 1 | 2 | 3 | 4 | 5 |  | 6 |
| 17 I am bothered by the slow recovery of my injury. | 1 | 2 | 3 | 4 | 5 |  | 6 |
| 18 I am afraid of being eliminated from competition because of poor performance. | 1 | 2 | 3 | 4 | 5 |  | 6 |
| 19 l am annoyed by my coach's bias against me. | 1 | 2 | 3 | 4 | 5 |  | 6 |
| 20 I am annoyed by my training load because it is too much for me. | 1 | 2 | 3 | 4 | 5 |  | 6 |
| 21 I am annoyed by my social skills because it seems like nobody likes me. | 1 | 2 | 3 | 4 | 5 |  | 6 |
| 22 I am annoyed with not getting along with my romantic partner. | 1 | 2 | 3 | 4 | 5 |  | 6 |
| 23 I am annoyed with communicating with my family. | 1 | 2 | 3 | 4 | 5 |  | 6 |
| 24 I worry about my academic skills because I do not know how to learn efficiently. | 1 | 2 | 3 | 4 | 5 |  | 6 |
|  |  |  |  |  |  |  |  |

Note: (a) items 1, 9, 17 represent "sports injury;" (b) items 2, 10. 18 represent "performance demand;" (c) items 3, I I, 19 represent "coach relationships;" (d) items 4, 12, 20 represent "training adaptation;" (e) items 5, 13, 21 represent "interpersonal relationships;" (f) items 6, 14, 22 represent "romantic relationships;" (g) items 7. 15, 23 represent "family relationships;" and (h) items 8, 16, 24 represent "academic requirements"

**Items and Instructions for Perceived Stress Scale (PPS)**

The questions in this scale ask you about your feelings and thoughts during the last month. In each case, you will be asked to indicate how often you felt or thought a certain way. Although some of the questions are similar, there are differences between them and you should treat each one as a separate question. The best approach is to answer each question fairly quickly. That is, don't try to count up the number of times you felt a particular way, but rather indicate the alternative that seems like a reasonable estimate.

For each question choose from the following alternatives:

**0. never; 1. almost never; 2. Sometimes; 3. fairly often; 4. very often**

1. In the last month, how often have you been upset because of something that happened unexpectedly?

2. In the last month, how often have you felt that you were unable to control the important things in your life?

3. In the last month, how often have you felt nervous and "stressed"?

4^a^. In the last month, how often have you dealt successfully with irritating life hassles?

5 ^a^. In the last month, how often have you felt that you were effectively coping with important changes that were occurring in your life?

6 ^a^. In the last month, how often have you felt confident about your ability to handle your personal problems?

7 ^a^. In the last month, how often have you felt that things were going your way?

8. In the last month, how often have you found that you could not cope with all the things that you had to do?

9 ^a^. In the last month, how often have you been able to control irritations in your life?

10 ^a^. In the last month, how often have you felt that you were on top of things?

^a^ , Scored in the reverse direction.

**Athlete Burnout Questionnaire (ABQ)**

| Item # | Subscale | Item Text |
| --- | --- | --- |
| 1 | RA | I'm accomplishing many worthwhile things in [sport] |
| 2 | E | I feel so tired from my training that I have trouble finding energy to do other things |
| 3 | D | The effort I spend in [sport] would be better spent doing other things |
| 4 | E | I feel overly tired from my [sport] participation |
| 5 | RA | I am not achieving much in [sport] |
| 6 | D | I don't care as much about my [sport] performance I used to |
| 7 | RA | I am not performing up to my ability in [sport] |
| 8 | E | I feel "wiped out" from [sport] |
| 9 | D | I'm not into [sport] like I used to be |
| 10 | E | I feel physically worn out from [sport] |
| 11 | D | I feel less concerned about being successful in [sport] than I used to |
| 12 | E | I am exhausted by the mental and physical demands of [sport] |
| 13 | RA | It seems that no matter what I do, I don't perform as well as I should |
| 14 | RA | I feel successful at [sport] |
| 15 | D | I have negative feelings toward [sport] |

Note: Response set is a 5-point Likert scale of (1) "almost never," (2) "rarely," (3) "sometimes," (4) "frequently," (5) "almost always." Items I and 14 are reverse-scored. RA

= reduced sense of accomplishment, E = emotional/physical exhaustion, D = devaluation.



Today:___________ date__________ month _________year

Name: _______________________ Age: _____years _____months

Gender (circle one): Male Female Nationality: __________

Current main sport: __________________

Name of current team (if applicable):______________________

Length of time competing in sport: ____ years ____months

Current performance status (circle one): Full-time Part-time

Current performance level (circle one):

| International | Senior national | Collegiate/University |
| --- | --- | --- |
| State/Regional | Junior national | County |
| Club | Other |  |
